# Supplementary material for: MetaRibo-Seq measures translation in microbiomes
Source: Nat Commun. 2020 Jun 29;11:3268. doi: 10.1038/s41467-020-17081-z (PMC7324362; doi:10.1038/s41467-020-17081-z)
Supplement: Supplementary file 10 — Supplementary Data 7 [file 41467_2020_17081_MOESM10_ESM.zip › File2/Confidence_High_Taxonomy/137556_out.krona.html]

Javascript must be enabled to view this page.

members
magnitude
magnitudeUnassigned
count
unassigned
taxon
rank

137556\_out

2

2
superkingdom
2

1239
phylum
2

186801
class
2

order
186802
2

2
family
186803

2
genus
33042

1
1262862
species

SRS017103\_contig\_number\_36802

1

SRS024331\_contig\_number\_6372
2293090
species
